# Predictors of Evolution Into Multiple Sclerosis After a First Acute Demyelinating Syndrome in Children and Adolescents

**DOI:** 10.3389/fneur.2018.01156

**Published:** 2019-01-15

**Authors:** Laura Papetti, Lorenzo Figà Talamanca, Alberto Spalice, Federico Vigevano, Diego Centonze, Massimiliano Valeriani

**Affiliations:** ^1^Neurology Unit, Multiple Sclerosis Center, Department of Neuroscience of Bambino Gesù Children's Hospital, Rome, Italy; ^2^Neuroradiology Unit, Imaging Department, Bambino Gesù Children's Hospital, Rome, Italy; ^3^Child Neurology Division, Department of Pediatrics, Sapienza University of Rome, Rome, Italy; ^4^Unit of Neurology and Unit of Neurorehabilitation, IRCCS Istituto Neurologico Mediterraneo (INM) Neuromed, Pozzilli, Italy; ^5^Center for Sensory-Motor Interaction, Aalborg University, Aalborg, Denmark

**Keywords:** multiple sclerosis, acute demyelinating event, pediatrics, clinically isolated syndrome, acute disseminated encephalomyelitis

## Abstract

**Background/Objective:** The aim of the study was to estimate the rate of evolution or for multiple sclerosis (MS), after a first acute demyelinating event (ADE) in pediatric patients, and to investigate the variables that predict this evolution.

**Methods:** We retrospectively evaluated the clinical and neuroradiological features of children who presented a first ADE between January 2005 and April 2017. All patients included underwent a baseline MRI, a cerebrospinal fluid and blood analysis, including virological examinations. The evolution into MS was determined by the 2013 International Pediatric Multiple Sclerosis Study Group (IPMSSG) criteria. Clinical and radiological features predictive of MS were determined using multivariate analyses.

**Results:** Ninety-one patients were selected (mean age at onset: 10.11 ± 4.6). After a mean follow-up of 5.6 ± 2.3 years, 35% of patients' conditions evolved to MS. In the logistic multivariate analysis of clinical and laboratory data, the best predictors of evolution into MS were: the presence of oligoclonal bands in CSF (*p* < 0.001), past infection with EBV (*p* < 0.001), periventricular lesions (*p* < 0.001), hypointense lesions on T1 (*p* < 0.001), and lesions of the corpus callosum (*p* < 0.001) including Dawson fingers (*p* < 0.001).

**Conclusion:** Our findings suggest that a pattern of neuroimaging and laboratory findings may help to distinguish between, at clinical onset, children with a monophasic syndrome (clinically isolated syndrome or acute disseminated encephalomyelitis) from those who will develop MS.

## Introduction

The term “acquired demyelinating syndrome” (ADS) is used to indicate the first clinical episode of acute CNS demyelination, which can either represent the sentinel attack of an underlying chronic demyelinating disorder or remain monophasic. Pediatric ADS occurs with an incidence of 0.5–1.66 per 100,000 children [(1–4); 37]. The proportion of ADS children who are ultimately diagnosed with MS ranges between 15 and 45% in different studies [(2, 3, 5, 6); 37]. An early differential diagnosis between a monophasic demyelinating inflammatory syndrome and multiple sclerosis is crucial, as increasing evidence favors early initiation of disease-modifying therapy [(2, 7–9); 38]. Early treatment avoids the accumulation of disability, delays the transition from “relapsing-remitting” into “secondary progressive MS,” and prevents axonal damage from occurring at an early stage (10, 11). Therefore, an early diagnosis of MS in children can slow down disease progression and reduce the level of future disability in adulthood (12). In recent years, studies have focused on defining criteria for early diagnosis in children and adolescents, as well as focusing on searching for predictive markers of progression into the recurrent disease [(2, 7–9); 37]. However, these studies have some limits: (1) the incidence of multiple sclerosis varies in different cohorts (15–46%), because different inclusion criteria are applied, even based on MRI findings alone, without considering clinical symptoms/signs [(2, 4, 5); 37; (13)]; (2) some studies include patients aged between 16 and 18 years, thus leaving childhood unexplored [37; (6)]; (3) it is difficult to distinguish between patients with ADEM from MS patients with ADEM-like onset, at the time of the first attack (6, 7, 9, 14); and (4) predictors with high specificity and positive predictive value show low sensitivity [(6, 15); 37]. The present study aims: (1) to analyze the clinical and neuroimaging features of a pediatric population at ADS onset; (2) to identify possible predictors of progression into MS; and (3) to compare the predictors obtained from our results with those of previous studies.

## Materials and Methods

### Participants and Inclusion Criteria

**Figure 1:**
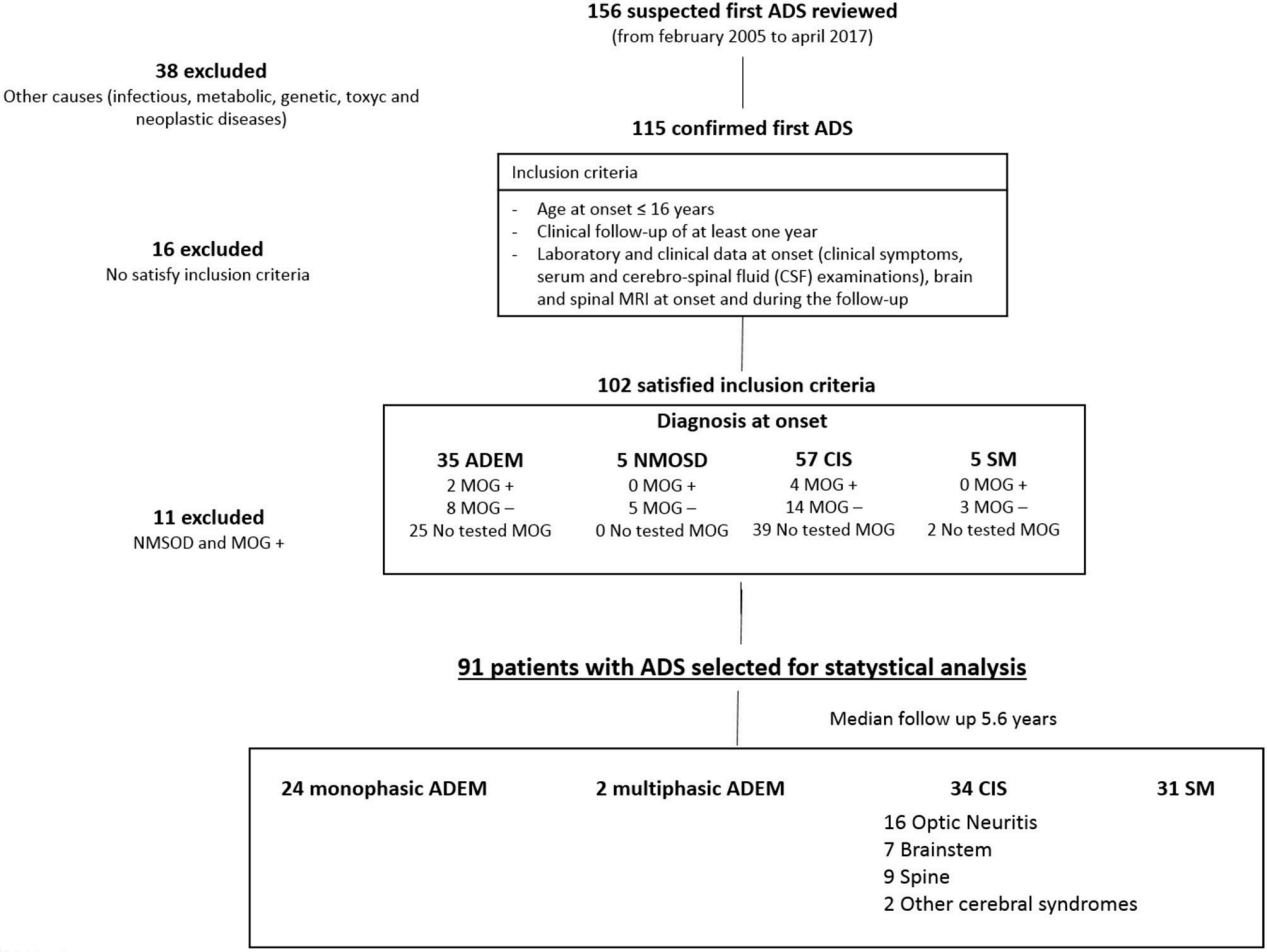
Study profile.

We performed a retrospective study that included all ADS patients that were referred to the Neurology Unit of the Bambino Gesù Children Hospital and the Department of Pediatrics of the Sapienza University, between January 2005 and April 2017. The records of a total of 156 patients with suspected ADS were reviewed (Figure 1). The inclusion criteria were: (1) a history of an acute neurological event suggestive of central nervous system (CNS) inflammatory demyelinating disease not attributable to other conditions (infectious, metabolic, neoplastic, congenital or vascular illness); (2) a clinical follow-up of at least 1 year; (3) age at onset ≤16 years; (4) available laboratory and clinical data at onset (clinical symptoms according to EDSS classification, serum, and cerebrospinal fluid (CSF) examinations, serology for EBV); and (5) brain and spinal MRI at onset and during follow-up.

Patients with secondary causes of demyelination (of toxic, genetic, metabolic, infectious, neoplastic), neuromyelitis optica spectrum disorders (NMOSD), ADS with serum positivity for myelin oligodendrocyte glycoprotein antibodies (MOGAbs) or a history of a progressive disease course were excluded.

In order to overcome the limitations of previously reported studies, we selected: (1) patients with CIS, ADEM, and MS diagnosed according to the 2013 International Pediatric Multiple Sclerosis Study Group (IPMSSG) criteria (16), which refers to the 2010 McDonald criteria for neuroradiological dissemination in time (DIT), and space (DIS) (9), (2) both patients with ADEM and CIS at onset; and (3) patients with a maximum onset age of 16 years.

The distinction between monophasic ADEM and multiphasic ADEM and MS with ADEM-like onset was made according to the 2013 IPMSSG criteria (16).

Diagnosis of NMOSD were made according to the “International consensus diagnostic criteria for neuromyelitis optica spectrum disorders” of 2015 (17). Patients with core clinical features of NMOSD (optic neuritis, acute myelitis or area postrema syndrome) were all tested for serum anti-aquaporin-4 antibodies (AQP4-ab).

The study was approved by the Ethical Committee of Bambino Gesù Children Hospital.

### Data Collection

The MRI, CSF, and clinical data were collected. An MRI was acquired using a 1.5T magnet or 3T magnet (Samsung), both available in two participating centers. We ensured that the brain MRI, performed at the onset, included axial and sagittal T2-weighted, fluid-attenuated inversion recovery (FLAIR)-weighted, T1-weighted MRI sequences, and T1-weighted MRI images after administration of gadolinium. All patients included in the study also underwent a spinal MRI at the onset of symptoms and before the start of corticosteroid therapy; dual-echo (proton-density and T2-weighted) conventional and/or fast spin-echo, STIR (as alternative to proton-density-weighted) and contrast-enhanced T1-weighted spin-echo (in case of presence of T2 lesions) sequences were acquired. The MRI scan revision was centralized and carried out by two operators (a clinician [LP] and a neuroradiologist [LFT]), blinded to clinical outcome, at the Bambino Gesù children's hospital. Lesion characteristics were recorded, including the location, distribution, border outline, symmetry, and number, as well as size and gadolinium capture. Tumefactive lesions were defined as such if larger than two cortical gyri. The presence or absence of black holes (non-enhancing hypointense lesions visible on T1-weighted sequences) and post-gadolinium enhancement were analyzed. Follow-up brain and spine MRI (performed after a minimum period of 3 months from baseline) were also reviewed in order to assess the neuroradiological dissemination in time (DIT) and space (DIS).

CSF examination data at onset included the cell count and the search for oligoclonal bands (OCBs). The presence of OCBs was determined by isoelectric focusing, combined with immunoblotting of matched serum, and CSF sample pairs. Virological assessments consisted of measuring serum viral antibodies (IgM and IgG by ELISA) and performing quantitative real-time PCR for the Epstein Barr virus (EBV).

The presence of MOGAbs was assessed only in patients that were observed since 2015, when we started performing MOGAbs detection by cell-based assays (CBAs).

### Statistical Analysis

Statistical analyses were completed using SPSS software (version 22.0). Descriptive statistics were used to compare the difference in frequencies in the MS group (clinical, laboratory, and MRI subtypes) and the non-MS groups (including CIS and ADEM). The Pearson's chi squared test for nominal categorical variables (e.g., sex, fever) and the Mann-Whitney *U*-test for continuous variables (e.g., age in months) were used. Logistic regression analyses were used to assess whether clinical, biochemical and MRI features of the initial attack could be predictors of evolution toward MS.

We used the multivariate logistic regression to build predictive models of evolution toward MS. As a first step, we selected the variables to test their single risk value (odd ratio-OR) in the univariate logistical analysis. Then, the variables that individually showed a significant OR (*p* < 0.05), were tested in the multivariate logistic analysis to build the models. In the multivariate logistic analysis, we used a backwards elimination process to test the correlation (*p* value cut-off = 0.10 for exclusion from the model) between variables, and calculated the sensitivity, specificity, positive predictive value (PPV), and negative predictive value (NPV) for each model built for the diagnosis of MS. Significance was fixed at *p* < 0.05. A multiple-comparison *post-hoc* correction was made with the Bonferroni correction, setting the significance cut-off at α/n with α = 0.05.

The current criteria available for pediatric age and those used in our models, were compared by using a univariable generalized linear model, with a logit link function, and a binomial error distribution.

## Results

During the considered time interval, only 102 out of the 156 patients initially identified were included in the analysis. The main reasons for exclusion from the study were: lack of follow-up data as to whether evolution into MS had taken place (25 patients), MRI exams that did not meet the requirements (lack of sequences, lack of spine MRI) (15 patients), secondary forms of demyelination (infections, genetic) (eight patients), and lack of laboratory data (six patients). Thirty-five patients presented features of ADEM, five with features of NMOSD, 57 with CIS, and five patients with features suggesting MS at onset.

Among 36 patients undergoing anti-MOGAbs detection, six (16.6%) showed positive results. Since anti-MOGAbs seem to have a protective role against MS development (18–20), none of the MOGAbs positive patients conditions evolved into MS and data from MOGAbs-positive patients and children with NMOSD were excluded from the statistical analysis, aimed at looking for factors predicting evolution into MS.

Ninety-one patients (42 females and 49 males; mean age at onset: 10.11 ± 4.60) who presented the first ADS in the observation period and met the remaining inclusion criteria were recruited (Figure 1). Onset diagnosis included 33 patients with ADEM, 53 patients with CIS, and five patients with MS at first ADS. The mean follow-up duration was 5.6 ± 2.3 years. During follow-up, the patients underwent clinical and MRI controls every 4–6 months during the first year of illness, and then with annual frequency. Patients with a normal MRI at onset and after 2 years, or those whose MRI became normal during the first 2 years of follow-up, without evolution to MS, discontinued MRI controls after 2 years and continued only a clinical follow-up. In case of a recurrence, the patient was clinically evaluated and underwent an MRI examination for the relapse. It is noteworthy to underline that the follow-up duration was similar in all patients, regardless of the clinical evolution.

### First ADS: Comparison Between ADEM and CIS Patients

At onset ADEM was diagnosed in 33 patients (36.3%), while 53 patients (58.2%) were classified as CIS. The main differences in clinical and neuroradiological features at onset between CIS and ADEM patients are summarized in Table 1. Five patients (5.4%) presented clinical and neuroradiological findings suggestive of MS at onset.

**Table 1:** Baseline clinical and demographic characteristics of ADEM and CIS patients.

**ADEM PATIENTS**		
Female sex (%)	42.4	
Age at onset (Mean age, SD)	7.7 ± 3.7 yrs	
History of recent infection (%)	63.6	
Fever at onset (%)	33.3	
Oligoclonal Bands (%)	15.2	
Evidence of past EBV infection (%)	48.5	
Pathological MRI (%)	100	
Evolution into MS (%)	21.2	
**CIS PATIENTS**		
Female sex (%)	45.3	
Mean age (Mean age, SD)	11.2 ± 3.5	
History of recent infection (%)	28.3	
Fever at onset (%)	11.3	
Oligoclonal Bands (%)	49.1	
Evidence of past EBV infection (%)	54.7	
Pathological MRI (%)	71.7	
**CIS topography:**		**Evolution into MS**
Optic Neuritis (n, %)	19 (35.8)	3/19 (15.7)
Brainstem	13 (24.6)	6/13 (46.1)
Spine	10 (18.9)	1/13 (7.7)
Other cerebral syndromes	11 (20.7)	9/11 (81.8)

In CIS patients, 18 patients (34%) clinically presented optic neuritis, 10 patients (18.9%) presented transverse myelitis, and 14 patients (20.8%) presented polyfocal symptoms, while 11 patients (20.8%) presented other monofocal symptoms. All ADEM patients presented encephalopathy. Other neurological signs or symptoms in ADEM patients, involved the pyramidal (57.6%), cerebellar (54.5%), the brainstem (33.3%), and the proprioceptive (30.3%) systems. The optic nerve was involved in 21.2% of ADEM patients, while 6.1% of patients showed sphincter dysfunction.

Patients with ADEM (mean age of 7.6 ± 3.7 years) were younger than those with CIS (mean age: 11.1 ± 3.5 years) (*p* < 0.001) and more often had a history of recent infection (63.6 vs. 28.3%; *p* < 0.005), fever at onset (33.3 vs. 11.3%; *p* < 0.05), and cerebellar symptoms/signs (54.5 vs. 11.3%; *p* < 0.001). Sensory disorders were more frequent in CIS than in ADEM patients (32.1 vs. 9.1%; *p* < 0.05). CSF pleocytosis was more frequent in ADEM than CIS (51.5 vs. 28.3%; *p* < 0.005) and OCBs were more frequently detected in CIS than in ADEM patients (49.1 vs. 15.2%; *p* < 0.001).

### Clinical Follow-up: MS vs. Non-MS Patients

Thirty-one patients (35%) evolved to MS, while the remaining 60 patients (65%) neither showed further demyelinating episodes nor neuroradiological relapses. While MS was diagnosed in seven out of 33 ADEM patients (21.2%) included in the analysis, 18 out of 53 patients with an initial diagnosis of CIS evolved to MS (34%). However, this difference was not significant (*p* > 0.05). Patients evolving to MS were more often female (66.7 vs. 33.3%; *p* < 0.01) and older (12.4 ± 3.4 vs. 8.9 ± 3.8; *p* < 0.01) in comparison with non-MS patients.

**Figure 2:**
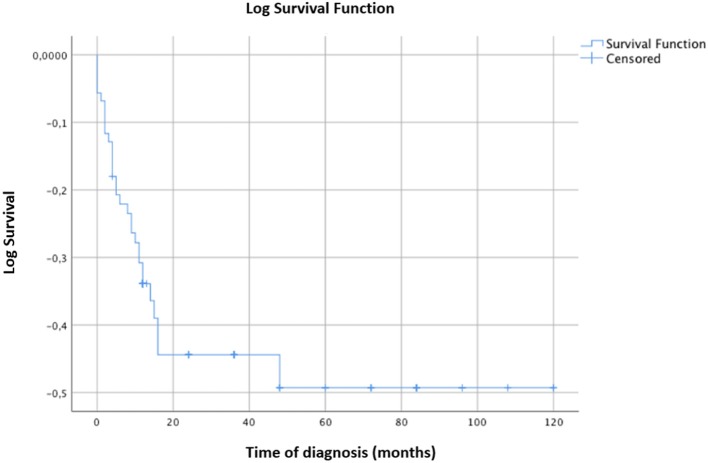
Kaplan-Meier survival analysis with event of interest “diagnosis of MS.” Censored patients (not converted into MS at last follow-up) are indicated on the curves. Time of diagnosis of MS is reported in months. The time axis is right-censored at 10 years. Evolution into MS occurred for most patients within 24 months from the onset and in no patient after 3.75 years.

**Figure 3:**
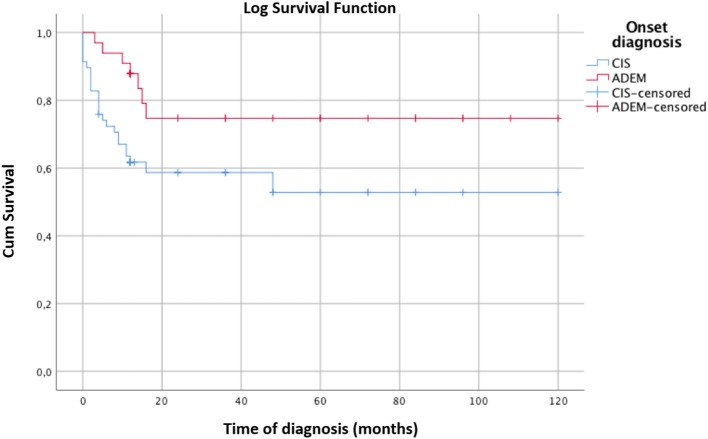
Kaplan-Meier survival analysis with event of interest “diagnosis of MS” comparing the survival curves of CIS and ADEM patients. Censored patients (not converted into MS at last follow-up) are indicated on the curves. Time of diagnosis of MS is reported in months. The time axis is right-censored at 10 years. The MS rate of evolution into MS is significantly faster for CIS than ADEM patients. However, both groups convert into MS within 24 months. Log rank test χ^2^ 4.97, *p* < 0.05.

The survival curve of the total patients is shown in Figure 2. The evolution to MS occurred mostly within 2 years of onset and did not occur in any patients after 3.75 years. CIS patients evolved to MS more rapidly than ADEM patients, but the final evolution times were equal for both groups (Figure 3).

CSF oligoclonal bands were positive in 73.3% of patients with MS (OR 9.23; *p* < 0.001) and there was evidence of past infection by EBV (IgG) in 86.7% of MS patients (OR11.52; *p* < 0.001) (Table 2). Interestingly, serum anti-EBV IgG were detected in 100% of the ADEM patients who evolved to MS, and only in 34.6% of monophasic ADEM (*p* < 0.001). On the contrary, a recent infection represented a protective factor for the evolution to MS (OR 0.18; *p* < 0.01).

**Table 2:** Onset clinical and neuroradiological features predictive of progression to MS (Univariate Analysis Logistics).

	**Frequency in MS vs. not MS (%)**	**Univariate analysis odds ratio (CI)**	**Sign. *p*-value**
**CLINICAL FEATURES**			
CIS	61.3 vs. 55 (*p =* 0.06)	2.61 (0.91–6.92)	0.06
ADEM	22.6 vs. 43.3(*p =* 0.06)	0.41(0.15–1.09)	0.056
Sex	F 66.7 M 33.3 vs. F 36.1 M 63.9 (*p* < 0.01)	3.54 (1.41–8.91) per female sex	**<0.01**
Age	12.4 ± 3.4 vs. 8.9 ± 3.8 (*p <* 0.01)	1.29 (1.12–1.48)	**<0.01**
Comorbidities	16.7 vs. 8.2 (*p =* 0.19)	2.24 (0.59–8.43)	0.23
Recent infection	16.7 vs. 63.2 (*p <* 0.01)	0.18 (0.06–0.53)	**<0.01**
Fever	3.3 vs. 26.2 (*p <* 0.01)	0.09 (0.01–0.77)	**<0.05**
Encephalopathy	26.7 vs. 42.6 (*p =* 0.15)	0.49 (0.18–1.27)	0.14
Pyramidal	53.3 vs. 44.3 (*p =* 0.44)	1.43 (0.59–3.46)	0.41
Brainstem	36.7 vs. 18.1 (*p =* 0.06)	2.63 (0.97–7.07)	0.055
PPT Sensitivity	36.7 vs. 16.4 (*p =* 0.03)	2.95 (1.08–8.07)	0.06
Proprioceptive	33.4 vs. 14.8 (*p =* 0.04)	2.88 (1.02–8.15)	0.06
Cerebellar	36.7 vs. 24.6 (*p =* 0.17)	1.77 (0.69–4.56)	0.23
Optic neuritis	16.7 vs. 41 (*p =* 0.17)	0.28 (0.09–0.85)	< 0.05
Bowel and Blurred	0 vs. 13.1 (*p =* 0.03)	0.01(0.00–0.01)	0.99
OGB present	73.3 vs. 23.1 (*p <* 0.01)	9.23 (3.37–25.23)	**<0.001**
Pleocytosis	40.2 vs. 41.3 (*p =* 0.55)	0.96 (0.39–2.34)	0.928
EBV IgM	0 vs. 8.2 (*p =* 0.128)	0.01 (0.00–0.01)	0.999
EBV IgG	86.7 vs. 36.1 (*p <* 0.01)	11.52 (3.55–37.32)	**<0.001**
EBV pcr	16.7 vs. 6.6 (*p =* 0.12)	2.85 (0.7–11.51)	0.142
**MRI FEATURES**			
Brain MRI with lesions	97.2 vs. 72.3 (*p =* 0.01)	9.13 (1.14-73.1)	**0.03**
**LOCATION**			
Periventricular	73.3 vs. 18 (*p <* 0.001)	12.5 (4.42–35.35)	**<0.001**
Subcortical	80 vs. 39.3 (*p <* 0.001)	6.17 (2.19–17.3)	**0.001**
Subtentorial	66.7 vs. 41 (*p =* 0.01)	2.88 (1.15–7.18)	**0.02**
Spine	43.3 vs. 29.5 (*p =* 0.14)	1.82 (0.73–4.52)	0.19
Deep WM	83.3 vs. 52.5 (*p =* 0.31)	4.53 (1.53–13.39)	**<0.01**
Cortical	3.3 vs. 21.3 (*p =* 0.02)	0.12 (0.01–1.02)	**0.05**
Corpus callosum	73.3 vs. 9.8 (*p <* 0.001)	25 (7.83–81.1)	**<0.001**
Internal capsule	13.3 vs. 16.4 (*p =* 0.26)	0.78 (0.22–2.74)	0.71
Basal Ganglia -Thalamus	0 vs. 23 (*p =* 0.02)	0.01 (0.00–0.01)	0.923
Brainstem	53.3 vs. 23 (*p <* 0.01)	3.8 (1.5–9.75)	**<0.01**
Cerebellar hemispheres	46.7 vs. 18 (*p <* 0.01)	3.2 (0.9–9.4)	0.071
Cerebellar peduncles	33.3 vs. 11.4 (*p =* 0.01)	2.88 (1.02–8.15)	**0.04**
Optic nerve	17.6 vs. 82.4 (*p =* 0.11)	0.37 (0.09–1.41)	0.147
Thalamus	10 vs. 25.4 (*p =* 0.27)	0.32 (0.08–1.23)	0.09
**DISTRIBUTION**			
Dawson Finger	43.3 vs. 1.6 (*p <* 0.001)	45.8 (5.59–376.19)	**<0.001**
Asymmetrical WM	93.3 vs. 65.6 (*p <* 0-005)	7.35 (1.59–33.89)	**0.01**
Symmetrical WM	60.1 vs. 39.3 (*p =* 0.05)	2.31 (0.94–5.64)	0.06
Asymmetrical GM	16.7 vs. 21.3 (*p =* 0.41)	0.64 (0.22–1.88)	0.42
Symmetrical GM	6.7 vs. 31.1 (*p <* 0.01)	0.15 (0.03–0.73)	0.05
**MORPHOLOGY**			
Poorly demarcated edges	46.7 vs. 67.2 (*p =* 0.03)	0.42 (0.17–1.04)	0.06
Well limited edges	83.3 vs. 29.5 (*p <* 0.001)	11.9 (3.95–36.12)	**<0.001**
Black holes	33.3 vs. 1.6 (*p <* 0.001)	30 (3.61–249.1)	**<0.01**
Tumefactive lesions	33.3 vs. 1.6 (*p =* 0.43)	0.83 (0.33–2.05)	0.692
Gad positive lesions	73.3 vs. 34.4 (*p <* 0.001)	5.23 (1.99−13.76)	**0.001**
T1 hypointhense lesions	66.7 vs. 4.9 (*p <* 0.001)	38.66 (9.66–154.72)	**<0.001**

In patients with MS diagnosis at onset (2 females and 3 males), 1 out of 5 cases (20%) clinically presented optic neuritis, 1 out of 5 cases (20%) with polyfocal symptoms in, and 3 out of 5 cases (33.3%) of monofocal symptoms. Four patients were aged over 16 and only one patient was younger. The MRI at the onset showed the presence of multifocal lesions. In particular, periventricular lesions were present in 5 out of 5 patients (100%), while juxtacortical lesions were present in 1 out of 5 cases (20%), and infratentorial lesions in 3 out of 5 cases (33.3%), and spine lesions in 3 out of 5 cases (33.3%). All five patients had a simultaneous presence of capturing and non-capturing gadolinium lesions at the onset MRI.

### MRI Lesions: MS vs. Non-MS Patients

In comparison with non-MS children and adolescents, in MS patients, MRI lesions were more frequently located in the periventricular (OR 12.5; *p* < 0.001) and subcortical (6.17; *p* = 0.001) regions, the corpus callosum (OR 25; *p* < 0.001), the brainstem (OR 3.8; *p* < 0.01), and the cerebellar peduncles (OR 2.88; <0.05). With regard to the distribution and morphology of the lesions, asymmetric WM lesions (OR 7.35; *p* = 0.01), lesions perpendicular to the corpus callosum known as “Dawson fingers” (OR 45.8; *p* < 0.001), lesions with well-limited margins (OR 11.9; *p* < 0.001), black holes (OR 30; *p* < 0.01), and gadolinium enhancing lesions (OR 5.23; *p* = 0.001) were more frequent in the MS group than in non-MS patients.

### MOGAbs Positive Patients

Among the 36 patients undergoing MOGAbs detection, six (16.6%) showed positive results. At the onset, these patients presented clinical MRI features of ADEM (2 patients, 33.3%), unilateral retrobulbar optic neuritis (RON) (one patient, 16.6%), bilateral RON (2 patients, 33.3%), and longitudinal extensive transverse myelitis (LETM) (one patient, 16.6%). No patient with a diagnosis of MS at onset showed positivity for MOGAbs. During the follow-up, no patient with positive anti-MOGAbs showed evolution to MS. One patient with ADEM presented a clinical and neuroradiological relapse which resulted in them being included in the multi-phase ADEM group. The remaining patients had a monophasic course.

### Models of ADS Evolution Into MS

In the multivariate logistic analysis (Table 3), the best combination of non-neuroradiological features predictive of progression into MS included: (1) the absence of a recent infection (OR 0.21, CI 0.05-0.79; *p* = 0.02), (2) OCBs in CSF (OR 8.34, CI 2.5-27.7; *p* = 0.001), and (3) past EBV infection (OR 11.47, CI 2.5-27.76; *p* < 0.001). However, the absence of a recent infection was excluded from the model after the Bonferroni correction. The definitive predictive model could predict evolution into MS with a sensitivity of 66, a specificity of 93, a positive predicting value (PPV) of 83, a negative predicting value (NPV) of 85, and an accuracy of 93.4%.

**Table 3:** Results of multivariate analysis.

**Model**	**Odds ratio (CI)**	***P* value**	**Sensibility, specificity, PPV, NPV, and accuracy**
**NOT NEURORADIOLOGICAL (ALL REQUIRED)**			
OCB IgG positivity in CSF	OR 9.4 (2.96–29.83)	0.001	66%; 93%; 83%; 85%; 93.4%
Serum EBV IgG positivity	OR 11.77 (3.18–43.24)	<0.001	
**1st NEURORADIOLOGICAL (ALL REQUIRED)**			
At least one Dawson Finger	OR 15.42 (1.13–209.73)	0.01	76.2%; 100%; 100%; 87.4%; 91%
At least one periventricular lesion	OR 12.42 (2.83–54.58)	<0.001	
At least one T1 hypointense lesion	OR 25.48 (4.5–144.22)	<0.001	
**2nd NEURORADIOLOGICAL (ALL REQUIRED)**			
At least one corpus callosum lesion	OR 8.87 (1.89–41.67)	<0.005	80%; 96%; 92%; 90%; 96.7%
At least one periventricular lesion	OR 11.64 (2.51–54.01)	<0.005	
At least one T1 hypointense lesion	OR 29.74 (4.91–180.16)	<0.001	
**LABORATORY AND NEURORADIOLOGICAL (ALL REQUIRED)**			
OCB IgG positivity in CSF	OR 4.8 (1.16–20.49)	<0.01	75%, 92%, 87%, 84.2%, 88.5%.
At least one periventricular lesion	OR 10.13 (2.26–45.33)	<0.01	
At least one T1 hypointense lesion	OR 45.34 (7.43–276.69)	<0.001	
^*^LABORATORY AND NEURORADIOLOGICAL (ALL REQUIRED)			
Serum EBV IgG positivity	OR 32.65, CI 8.36–231.76	<0.001	60%, 100%, 100%, 89.6%, 90.9%.
At least one periventricular lesion	OR 4.5, CI 1.23–16.72	<0.01	
At least one T1 hypointense lesion	OR 25.18 CI 6.56–135.87	<0.001	

* *Only for MS with ADEM like onset*.

With regard to the MRI data, we found that an abnormal brain MRI was associated with a higher risk of evolution into MS (97.2% of patients) than a normal brain MRI (e.g., isolated optic neuritis or myelitis) (OR 9.13, *p* < 0.05). The most important MRI baseline features for predicting evolution into MS were: (1) at least one Dawson finger (OR 15.42, CI 1.13-209.73; *p* = 0.01), 2) at least one periventricular lesion (OR 12.42, CI 2.83-54.58; *p* < 0.001), and (3) at least one T1 hypointense lesion (OR 25.48, CI 4.5-144.22; *p* < 0.001). The model showed a sensitivity of 76.2, a specificity of 100, a PPV of 100, an NPV of 87.4, and an accuracy of 91%. A second MRI model that combined good sensitivity and specificity included: (1) at least one periventricular lesion (OR 26.82, CI 2.64-271.98; *p* = 0.005), (2) at least one subcortical lesion (OR 13.17, CI 1.3-133.2; *p* < 0.05), (3) at least one lesion of the corpus callosum (OR 9, CI 1.6-0.84; *p* = 0.01), and (4) at least one T1 hypointense lesion (OR 17.54, CI 2.57-119.72; *p* = 0.003). However, the variable “at least one subcortical lesion” was removed from the model after the Bonferroni correction. The definitive model showed a sensitivity of 80, a specificity of 96, a PPV of 92, an NPV of 90, and an accuracy of 96.7%.

The multivariate analysis of the combined non-neuroradiological and MRI findings, showed that the MRI features that best distinguished patients evolving into MS, from those that would not evolve into MS were: (1) at least one periventricular lesion (OR 10.13, CI 2.26-45.33; *p* < 0.01), (2) at least one T1 hypointense lesion (OR 45.34, CI 7.43-276.69; *p* < 0.001), and (3) the presence of OCBs (OR 4.8, CI 1.16-20.49; *p* < 0.01). This model showed a sensitivity of 80, a specificity of 92, a PPV of 87, an NPV of 84.2, and an accuracy of 88.5%.

The results of the comparison between the current MRI criteria and our neuroradiological models are featured in Table 4.

**Table 4:** Current criteria compared with our models in our cohort.

**Criteria**	**KIDMUS 2004**	**McDonald2005 (Barkhof)**	**Callen per MS 2009**	**Callen MS vs. ADEM 2009**	**McDonald 2010 DIS**	**Verhey 2011**	**I model**	**II model**
Features	Two of two: -lesions perpendicular to the long axis of the corpus callosum - well-defined lesions	Three of four: ≥9 T2 lesions o 1 gad + lesion ≥3 periventricular ≥1 subtentorial ≥1 subcortical	Two of three: ≥5 T2 lesion ≥2 periventricular ≥1 brainstem	Two of three -no diffuse bilateral lesions - black holes -≥1 brainstem lesion	Two of four ≥1 periventricular ≥1 subcortical ≥1 subtentorial ≥ 1spine	Two of two: ≥1 periventricular ≥1 T1 hypointhense	Dawson Finger ≥1 periventricular lesion ≥1 T1 hypointhense lesion	≥1 Periventricular lesion ≥1 Corpus callosum lesion ≥1 T1 hypointhense lesion
Sensibility	21–47%	56–91%	26–74%	95%	85–100%	70–84%		
95% CI	**38.5%**	**56%**	**45%**	**16%**	**85%**	**80%**	**76%**	**80%**
Specificity	98-100%	30-100 %	68-100%	90%	80–86%^*^	90–93%		
95% CI	**100%**	**85%**	**90%**	**100%**	**61%**	**91.4%**	**100%**	**96%**
PPV	82–100%	34–69%	37-97%	71%	76%^*^	76%		
95% CI	**100%**	**70%**	**60%**	**100%**	**58%**	**85%**	**100%**	**92%**
NPV	61–87%	40–98%	90–91%	99%	100%^*^	96%		
95%CI	**74%**	**77%**	**70%**	**76%**	**88%**	**91%**	**87%**	**90%**

*Bold figures refer to criteria applied to our cohort*.

* *Children under the age of 12*.

## Discussion

This is the first study conducted on a population of Italian ADS patients, with the aim of finding predictive factors of evolution into MS. We found that the MS risk is high in CIS and ADEM patients, whose MRI shows lesions of the corpus callosum and of the periventricular regions, T1 hypointense lesions, and whose clinical data reveal the presence of OCBs, and a past infection by EBV.

In comparison with previous findings [37; (5–7, 9, 14)], the present study has the following strengths: (1) the use of the recent IPMSSG 2013 criteria for the diagnosis of MS and other demyelinating disorders, (2) the exclusive inclusion of early onset MS patients (maximum age of 16 years at onset), and (3) the research into MS predictors valid for both patients with ADEM and CIS-like onset.

In the pediatric age range, MS can be difficult to predict at the time of the first ADS, especially in very young children. In 2007, the IPMSSG proposed provisional definitions for pediatric acquired demyelinating disorders of the CNS (21). The revised McDonald criteria deals with MS diagnosis in children on the basis of studies providing further insight into clinical, CSF and MRI features (9). However, all versions of the McDonald diagnostic criteria are based on studies in patients with MS onset in adulthood. Although the diagnostic ability of the 2010 McDonald criteria in children is similar to that in adults (9, 22, 23), caution must be taken when applying the 2010 criteria to children aged younger than 11 years (9). Considering these limits, in 2013 the IPMSSG decided to revise the diagnostic criteria in order to facilitate the diagnosis of inflammatory demyelinating diseases in children (16).

### Can ADEM Evolve Into MS?

Using the IPMSSG 2013 criteria in our patients, we found that the overall risk of MS after an initial inflammatory demyelinating event was 35%, in line with data reported in the previous case series (21–57%) [(1–3); 37, 38].

Our results show that the onset phenotype (ADEM or CIS) did not determine the risk of evolution to MS. In particular, in our ADEM patients, the risk of progression to MS was 21.2%, similar to that reported in previous studies (10–29%) [38; (1–3, 24)]. This means that not only patients with CIS but also those with a first ADEM-like ADS can develop MS. Our findings support the recent concept of a partial overlap between ADEM and MS, especially in pediatric patients. The IPMSS 2013 criteria focusses on this overlap, considering the possibility of a diagnosis of MS after a first episode of ADEM (24).

### Clinical, Laboratory, and MRI Predictors

Concerning the evolution to MS, we found that a recent infection represented a protective factor (OR 0.124; *p* = 0.01), since most of the patients with a recent infection (63.2%) did not evolve to MS. In most cases, the infections involved the upper respiratory or gastrointestinal tract, and were associated with viral or bacterial agents that could not be identified by means of serological tests. The protective role of a recent infection in patients with ADS has also been described in a previous study (4). We can therefore hypothesize that, in patients with a recent infection, demyelinating lesions are probably due to a transient, and self-limited post-infectious immunological response (25).

In the present study, previous infection with EBV, documented by serum IgG positivity, was associated with an increased risk of progression to MS (OR 11.52; *p* < 0.001). In particular, serum anti-EBV IgG positivity was detected in 100% of ADEM patients evolving to MS, and in only 34.6% of ADEM patients with monophasic disease (*p* < 0.001). Previous studies have shown that serum anti-EBV IgG is more frequently found in children with MS (85–88%) than in healthy individuals (44–77%) (26–29). The role of EBV in the pathogenesis of MS in not clearly understood, but several hypotheses have been proposed (30, 31). MS may be the result of the ability of EBV to establish a persistent brain infection in persons predisposed to develop the disease, allowing EBV-infected B cells to accumulate in the CNS (30). A deficiency in CD8+ effector memory T cells, reported in MS patients (31), may explain a possible impairment in EBV infection control (32).

Our results underline the importance of OCB detection in children and adolescents with a first ADS. In our patients, OCB positivity in the CSF was an important predictor of the evolution to MS (OR 9.23; *p* < 0.001). Although the presence of OCB is not pathognomonic for MS, as OCBs are detected in 8–15% of ADS children who will not develop MS [(2, 24); 38], the presence of OCB in the CSF should be considered a strong predictor of MS evolution (33). In adult MS patients, the presence of oligoclonal bands increases the risk of clinically definite multiple sclerosis [adjusted hazard ratio 1.3 (1.0–1.8)] and of disability [adjusted hazard ratio 2.0 (1.2–3.6)], regardless of other factors (34). In a recent pediatric prospective study, OCBs were positive in 70% of MS patients (18). While OCB positivity was excluded from the 2010 criteria, in the latest 2017 version of the McDonald criteria, the presence of OCBs is considered a criterion for DIT (35).

Previous studies have shown that anti-MOGAbs can be found in pediatric patients with ADEM and NMOSD (20) and have a protective influence against developing MS (18–20). In our study, anti-MOGAbs were found in 16.6% of patients tested, none of which presented an evolution to MS.

**Figure 4:**
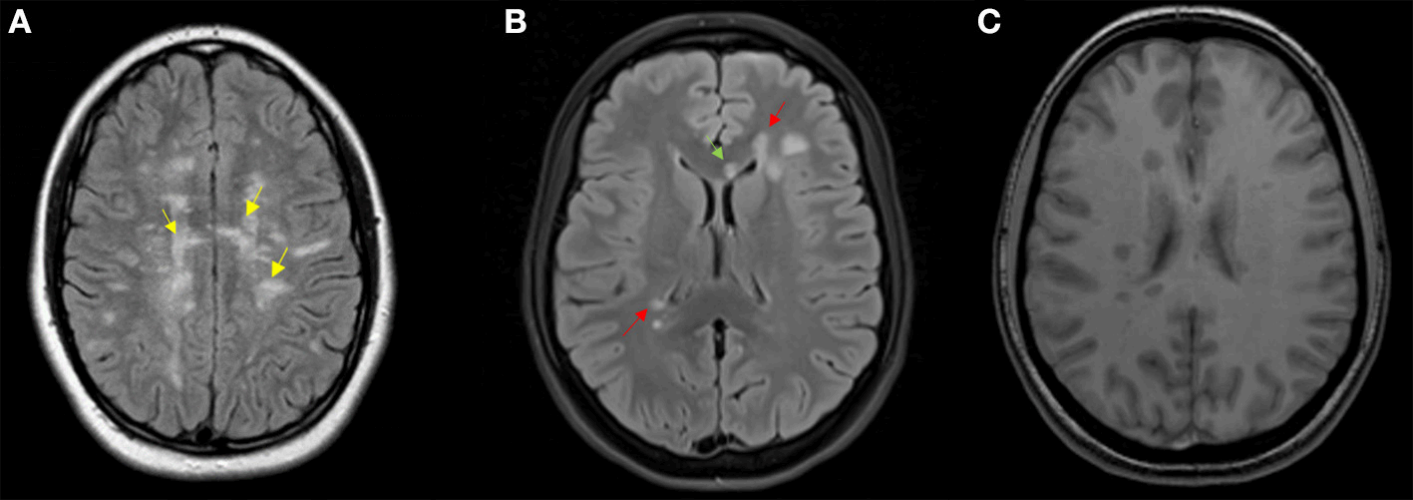
Representative MRI scans. **(A)** Axial T2 Flair MRI of a 15-year-old girl featuring Dawson's finger lesions (yellow arrows); **(B)** axial T2 Flair MRI, and **(C)** axial T1 MRI of a 16-year-old girl showing periventricular (red arrows) and corpus callosum (green arrow) lesions, and T1 hypointense lesions.

Considering the brain and spine MRI at the onset in our cohort, neuroradiological features that showed the highest specificity with the highest predictive value of evolution into MS included: (1) at least one Dawson finger, (2) at least one periventricular lesion, and (3) at least one hypointense lesion on T1 (Figure 4). However, this model had low sensitivity (76.2%), probably because of a low incidence of Dawson fingers in pediatric MS, in particular at the onset of the disease (36). Dawson fingers, attributed to inflammation around the long axis of medullar veins, are considered highly specific for multiple sclerosis (36), so they are included in the KIDMUS (Kids with Multiple Sclerosis) criteria (37). Recently, a large prospective study found that one or more T1 hypointense lesions and T2 periventricular lesions are strongly associated with a diagnosis of MS in children (18).

In order to obtain good sensitivity and specificity, we built a second model that included: (1) at least one periventricular lesion, (2) at least one lesion of the corpus callosum, and (3) at least one T1 hypointense lesion (Figure 4). This model showed a specificity of 96, a sensitivity of 80, and an accuracy of 96.7%. While the presence of periventricular lesions and T1 hypointense lesions have already been included in a previous study (5), lesions of the corpus callosum, regardless of their morphology, have never been considered in the previous pediatric diagnostic criteria. Our data are supported by a study showing a predictive value of corpus callosum lesions (HR 0.16 CI 0.03-0.89; *p* = 0.04) (4).

The main limitation of our study is represented by its retrospective design. In particular, this negatively affected the total number of recruited patients. However, in order to obtain reliable results, we analyzed variables that could be available in all patients. Future prospective studies will hopefully validate the predictive models proposed in this paper.

### Conclusions

This is the first study to assess the risk of evolution into MS in an Italian pediatric population at a first ADS. In comparison with the 2010 McDonald criteria, both neuroradiological models proposed in our study have the advantage of being independent of the age at onset and of ADEM-like onset. Moreover, our first neuroradiological model showed the same high specificity and PPV as KIDMUS (100%), but had better sensitivity (76.2 vs. 38%). The sensitivity was increased further in our second model (80%), which maintained a high specificity (96%).

## Author Contributions

LP is responsible for the study concept and design, and performed data acquisition. LF collects and analyzes the MRI data. AS contributed to data acquisition. DC and FV conducted the critical revision of the manuscript for intellectual content. MV performed the analysis and interpretation of data, and conducted the study supervision.

### Conflict of Interest Statement

The authors declare that the research was conducted in the absence of any commercial or financial relationships that could be construed as a potential conflict of interest.
